# Adolescent cannabidiol treatment produces antidepressant-like effects without compromising long-term cognition in rats

**DOI:** 10.1007/s43440-025-00750-5

**Published:** 2025-06-16

**Authors:** Laura Gálvez-Melero, Itziar Beruete-Fresnillo, Sandra Ledesma-Corvi, M. Julia García-Fuster

**Affiliations:** 1 Present Address: IUNICS, University of the Balearic Islands, Cra. de Valldemossa km 7.5, Palma, E-07122 Spain; 2 Health Research Institute of the Balearic Islands (IdISBa), Palma, Spain; 3 Department of Medicine, University of the Balearic Islands, Palma, Spain; 4 Red de Investigación en Atención Primaria en Adicciones (RIAPAd), ISCIII, Madrid, Spain

**Keywords:** Biological sex, Adolescence, Antidepressant, Cannabidiol, Hippocampus, Neuroplasticity

## Abstract

**Background:**

Recent preclinical studies have shown sex-dependent antidepressant-like responses of cannabidiol in adolescence, which were dependent on biological sex, early-life stress, and dose. In particular, cannabidiol (10 mg/kg) induced acute and sustained antidepressant-like responses in adolescent male rats, while it lacked efficacy in females. This follow-up study aimed at further characterizing cannabidiol’s effects in adolescence, in an attempt to overcome female unresponsiveness, while also evaluating its long-term safety profile in adulthood.

**Methods:**

Groups of adolescent rats of both sexes were treated (*ip*) with cannabidiol (10, 30, 60 mg/kg) or vehicle (1 ml/kg) for 7 days. Acute (30 min post-injection) and repeated (24 h post-treatment) antidepressant-like responses were measured in the forced-swim test. Brains were collected to evaluate several neurochemical correlates in the hippocampus (CBR1, CBR2, BDNF, and cell proliferation) after adolescent cannabidiol exposure (acute and repeated). Some rats were left undisturbed until adulthood, when long-term effects on cognition were measured in the Barnes maze (short- and long-term memory) or affective-like responses in the forced-swim test. Data was analyzed with two-way ANOVAs (independent variables: sex and treatment).

**Results:**

While the dose of 10 mg/kg of cannabidiol induced antidepressant-like effects in adolescent rats, higher doses had no effect in adolescent rats of both sexes. No changes were observed in any of the hippocampal neuroplasticity markers evaluated. Adolescent cannabidiol exposure did not induce long-term changes in cognitive performance or affective-like behavior.

**Conclusions:**

Overall, our data suggest that adolescent cannabidiol treatment produces dose-dependent antidepressant-like effects of moderate magnitude without compromising long-term cognition in rats.

**Supplementary Information:**

The online version contains supplementary material available at 10.1007/s43440-025-00750-5.

## Introduction

Given the pressing need for new fast-acting antidepressants for treatment-resistant depression in adolescence, our group has focused its attention on characterizing pharmacological agents at the preclinical level while incorporating sex as a biological variable (for an extended introduction on this topic see our recent review; [1]). In this context, cannabidiol, a nonpsychomimetic cannabinoid extracted from the most popular recreational and medicinal plant *Cannabis sativa*, has shown a great therapeutic potential on improving diverse affective-like symptomatology (e.g., [2–9]). However, the efficacy of cannabidiol is known to be dependent on some variables such as strain and/or species used, biological sex, age, prior stress and/or treatment duration, dose, and/or route of administration (reviewed by [1]).

In general, besides prior reports in the literature mainly centered in adult rodents which have been included in several reviews (see [1–3] and references within), several studies from our group evaluated the effects of cannabidiol in adolescence at the preclinical level by utilizing distinct behavioral tests used to measure antidepressant-like responses (i.e., forced-swim, novelty-suppressed feeding, open field, sucrose preference; [4, 5, 10]). First, we compared the effects of a dose-response treatment with cannabidiol (3, 10, and 30 mg/kg) in adolescent vs. adult male naïve rats [4]. In particular, the results showed that a repeated 7-day treatment with cannabidiol was efficacious in adolescence and adulthood, but with different doses (10 mg/kg for adolescence and 30 mg/kg for adulthood) and with a different length in response (2 days vs. 21 days post-treatment in adolescence and adulthood, respectively), showing a diminished response at younger ages [4]. Then, we performed a separate study in adolescent rats to ascertain the potential fast-acting and long-term antidepressant-like responses of the dose of 10 mg/kg of cannabidiol while considering sex as a biological variable by including rats of both sexes, and utilizing maternal deprivation as a model of early-life stress [10]. The main results extended the previously observed effects in adolescent male rats [4], to show a rapid and sustained antidepressant-like response in naïve rats, while proving the need for higher doses of cannabidiol when rats had been stressed early in life [10]. Contrarily, and aligned with prior studies in adult female rodents (e.g., [3]), cannabidiol (10 mg/kg, 7 days, 1 dose/day) lacked efficacy in adolescent female rats [10]. Against this background, this follow-up study aimed at evaluating whether higher doses of cannabidiol (30 or 60 mg/kg) could be efficacious in the previously unresponsive adolescent female rats.

Even though cannabidiol is already approved for the treatment of certain clinical uses, such as a rare type of pediatric epilepsy (Epidiolex^®^, pure cannabidiol), its mechanism of action is not fully elucidated. It is hypothesized to display a complex pharmacological profile with a broad range of molecular targets (e.g., [11]; recently reviewed by [12]); for example, cannabidiol acts as a negative allosteric modulator with low affinity to the cannabinoid receptors (CB1R and CB2R; [13–16], inhibits the fatty acid amide hydrolase (i.e., increasing anandamide; [17]), but also interacts with 5-HT1A receptor (e.g., [18]), among many systems (reviewed by [11] and more recently by [1]). In the context of potential molecular events taking place in the hippocampus that might be associated with the antidepressant-like response of cannabidiol in adolescence, we aimed at evaluating CB1R and CB2R (reviewed by [12]), and/or the activation of neurotrophic factors such as BDNF (reviewed by [19]), or the regulation of the early stage of hippocampal neurogenesis (e.g., rates of cell proliferation; [20, 21]). The induction of such markers of hippocampal neuroplasticity has been described as key transducers of antidepressant effects. In fact, and as reviewed by Osborne and colleagues [22], cannabidiol may promote neurogenesis by increasing BDNF levels, changes in which may correlate with improved functional outcomes in behavior, and particularly for that study in cognition.

Finally, while cannabidiol has not been associated with dependence after its repeated use [23] or toxicity in animals and humans [11], and even proved to ameliorate cognition in multiple preclinical models of cognitive impairment, including models of neuropsychiatric disorders (reviewed by [22, 24]), there is a lack of studies evaluating the long-term impact of repeated cannabidiol exposure during adolescence later on in adulthood. Therefore, the present study checked for potential prolonged effects on affective-like behavior or cognitive performance induced by adolescent cannabidiol exposure (10, 30, and 60 mg/kg, 7 days) in rats of both sexes. Overall, the present study aimed at characterizing the antidepressant-like effects and drug targets of adolescent cannabidiol exposure, as well as the long-term effects emerging in adulthood.

## Materials and methods

### Animals

A total of 212 Sprague-Dawley adolescent rats (109 males and 103 females) that were bred in the animal facility at the University of the Balearic Islands were used in this study. Rats were initially housed in groups of 3 to 4 rats, and later on, as they size increased, separated into groups of 2 following regulations on the number of rats each cage could hold by size. Rats were housed in standard cages under controlled environmental conditions (22 °C, 70% humidity, and 12 h light/dark cycle, lights on at 8:00 AM) and with access to *ad libitum* tap water and a standard diet. All animal experiments complied with the ARRIVE guidelines [25] and the EU Directive 2010/63/EU of the European Parliament and the Council, and were previously approved by the Local Committee (University of the Balearic Islands; CEEA: 155-12-20) and the regional Government (Conselleria de Medi Ambient, Agricultura, Pesca i Alimentació, Direcció General Agricultura, Ramaderia i Desenvolupament Rural, Govern de les Illes Balears Exp.: 2021/05/AEXP). All efforts were made to reduce the number of rats and their suffering, and in this context, to avoid unnecessary stress in females, the particular phases of the estrous cycle were not assessed across time (see similar results from our group: [10, 26]). This is supported by the fact that both sexes appeared similarly variable due to hormonal periodicity [27, 28], in line with the parallel ranges of variability observed for each behavioral measure analyzed independently of biological sex, and together with the fact that cyclicity was not a variable in our research question [29].

### Cannabidiol treatment during adolescence

**Fig. 1:**
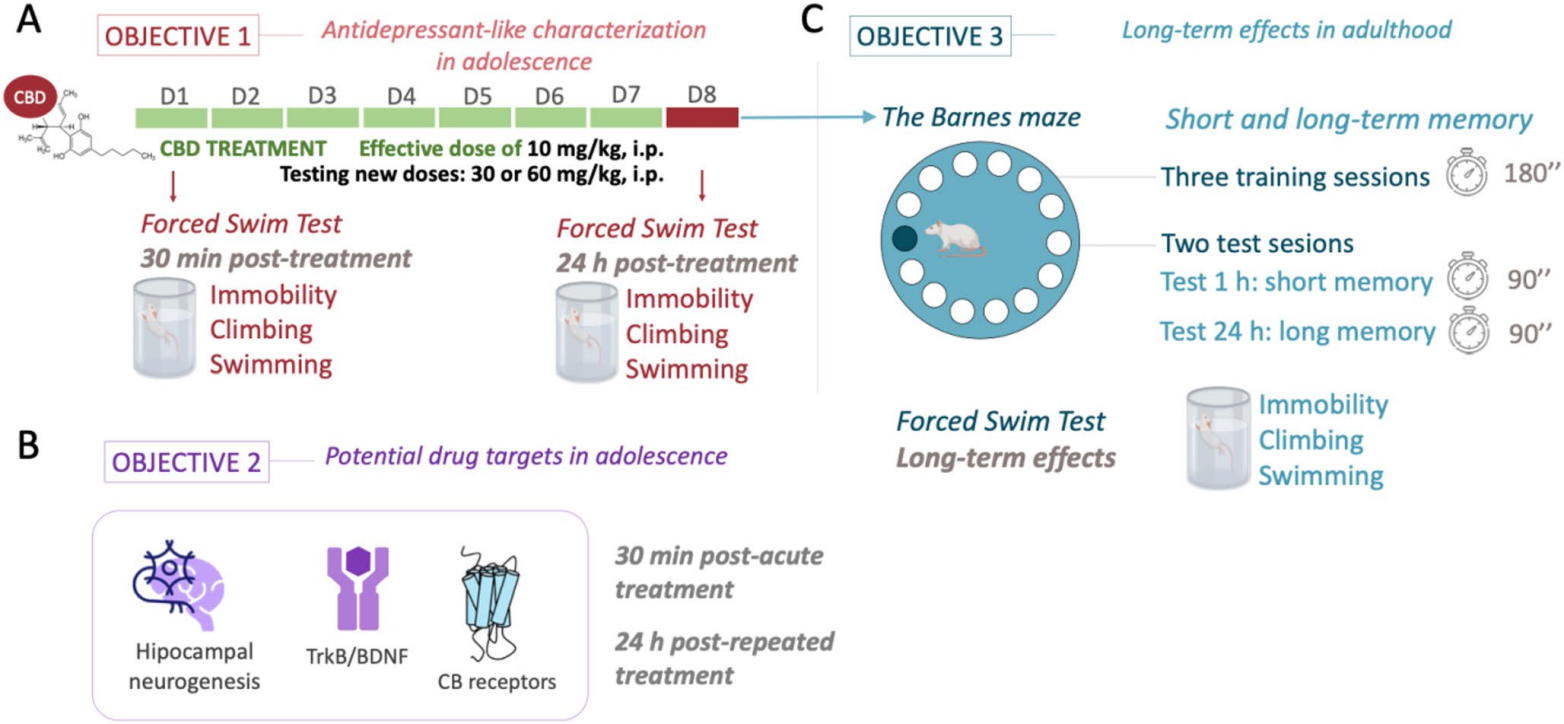
Experimental design. **(A)** Objective (1) Characterization of the antidepressant-like response of cannabidiol (CBD) as measured under the stress of the forced-swim test (FST) in adolescent rats of both sexes. Doses tested: 10, 30, and 60 mg/kg, *ip*; acute effects as measured 30 min post-acute dose; repeated effects as measured 24 h after 7 doses (1 dose/day). **(B)** Objective (2) Potential drug targets: neurochemical markers evaluated in the hippocampus of adolescent rats of both sexes following CBD treatment. Doses tested: 10, 30, and 60 mg/kg, *ip*; 30 min post-acute treatment and 24 h post-repeated treatment. Cell proliferation (i.e., Ki-67), cannabinoid (CB) receptors, and BDNF (brain-derived neurotrophic factor) as a neuroplasticity marker. **(C)** Objective (3) Long-term effects in adulthood. Short- and long-term memory in the Barnes maze and behavioral despair in the FST

The experimental design consisted of different sets of experiments with groups of randomly allocated rats to ascertain the 3 Objectives detailed in Fig. 1. For Objective 1, centered in characterizing the antidepressant-like response of cannabidiol under the stress of the forced-swim test, we first used a group of adolescent rats (a total of 48 males and 28 females) treated with 10 mg/kg (*ip*) of cannabidiol either acutely or repeatedly (7 days, 1 dose/day) [4, 5, 10]. This was later complemented with new doses of cannabidiol (30, 60 mg/kg) [2, 3], which were tested in 22 male and 23 female adolescent rats (i.e., acute and repeated effects). For Objective 2, we performed a separate experiment with 39 male and 52 female adolescent rats that were not exposed to behavioral testing to ascertain putative drug targets of cannabidiol administration during adolescence. Finally, a subset of rats from Objective 1 were left undisturbed until adulthood to assess the long-term effects of cannabidiol (Objective 3). For all experiments, rats were handled prior to any drug treatment, and were exposed to a daily dose of cannabidiol (10, 30, or 60 mg/kg, *ip*) (purity ≥ 98%; THC Pharm, Germany) for 7 days during mid-adolescence. The doses of cannabidiol were selected based on our own prior studies (e.g., [4, 5, 10]) and following prior ones performed in rodents (reviewed by [11]). Control rats received vehicle solution (1 ml/kg of DMSO) at the same treatment times.

### Antidepressant-like characterization in adolescence

The antidepressant-like response of cannabidiol was ascertained during adolescence in the forced-swim test, which is an accepted experimental tool to score drugs for antidepressant activity under a stressful situation [30, 31]. We selected this test as compared to others (i.e., novelty-suppressed feeding, sucrose preference), since our prior studies with cannabidiol proved greater measurements of efficacy, particularly for the forced-swim test (e.g., [4, 10]). Therefore, and following standardized procedures in our group (e.g., [10, 26]) rats from Objective 1 (Fig. 1) were placed in individual tanks (41 cm high x 32 cm diameter) filled with water (25 ± 1 °C; depth of 25 cm) for a 15 min pre-test session during which they could realize that escaping was not possible. The acute and repeated antidepressant-like potential of cannabidiol was evaluated through 5-min test sessions across time (30 min post-acute and 24 h post-repeated treatments). Sessions were videotaped, and the time spent immobile vs. in active behaviors (climbing or swimming) was scored for each rat (Behavioral Tracker software, CA, USA) by an experimenter blinded to the treatment groups. We have previously demonstrated that the potential effects of learning due to this test repetition can be controlled by applying the same conditions to all rats across time, therefore obtaining consistent responses across testing days (i.e., [4, 32, 33]).

### Potential drug targets in adolescence

Adolescent rats from Objective 2 (Fig. 1), which were not exposed to behavioral testing, were euthanized by rapid decapitation 30 min post-acute or 24 h post-repeated cannabidiol treatments (10, 30, 60 mg/kg, *ip*, 1 or 7 doses, respectively) [10]. For each rat, brain tissue was collected as follows; the right hippocampus was freshly dissected and fast-frozen in liquid nitrogen, and the left half-brain was quickly frozen in -30 °C isopentane [4, 5]. All samples were then stored at -80 °C until further use.

#### Western blot analysis

The right hippocampus from all groups (acute and repeated samples) was homogenized following standard procedures to later evaluate the protein content of BDNF and cannabinoid receptors (CB1R and CB2R) through western blot [33, 34]. A total of 40 µg of homogenate from each sample was separated by electrophoresis through 10–14% SDS-PAGE mini-gels (Bio-Rad Laboratories, CA, USA), transferred to nitrocellulose membranes, and incubated overnight at 4 °C with the specific primary antibodies (anti-BDNF, dilution 1:10000 and anti-CB1, dilution 1:2000, both from Abcam, Cambridge, UK; anti-CB2, dilution 1:1000, Cayman Chemical, Ann Arbor, MI, USA). The next day, membranes were incubated with the proper secondary antibody linked to horseradish peroxidase (1:5000 dilution; Cell Signaling), ECL reagents (Amersham, Buckinghamshire, UK), and exposed to an autoradiographic film (Amersham ECL Hyperfilm) for 1–60 min. Films were then analyzed by densitometric scanning (GS-800 Imaging Calibrated Densitometer, Bio-Rad). The quantification procedure was evaluated for each sample at least 3 to 6 times in different gels (each gel contained different brain samples from control and cannabidiol-treated rats). Percent changes in immunoreactivity were estimated with respect to male control samples in the various gels (100%), and the mean value was used as a final estimate. Membranes were then stripped and reprobed with ß-actin (clone AC-15) (1:10000; Sigma–Aldrich, MO, USA), which was used as a loading control, since the treatments evaluated did not alter it.

#### Immunohistochemical analysis

The hippocampal extent (-1.72 to -6.80 mm from Bregma) of rats exposed to repeated cannabidiol was cryostat-cut in 30 µm sections, slide-mounted, and stored at -80 °C until hippocampal cell proliferation (i.e., all diving cells within a cell cycle were labeled with Ki-67) was evaluated through immunohistochemistry [35, 36]. In particular, Ki-67 labeling was evaluated in 3 slides per animal containing 8 hippocampal tissue Sect. (24 sections total; every 8th section throughout the entire extent of the hippocampus), which were post-fixed in 4% paraformaldehyde for 1 h. Later on, slides were exposed to several steps such as antigen retrieval, blocking in peroxidase solution and BSA (with 1% serum and 0.05% Triton X-100), followed by an overnight incubation with rabbit polyclonal anti-Ki-67 (1:40000; kindly provided by Drs. Huda Akil and Stanley J. Watson, University of Michigan). The next day, slides were incubated with biotinylated anti-rabbit secondary antibody 1:1000 (Vector Laboratories) for 1 h, followed by incubations with an Avidin/Biotin complex (Vectastain Elite ABC kit; Vector Laboratories), the chromogen 3,3’-diaminobenzidine (DAB), and counterstaining with cresyl violet. Finally, tissue was dehydrated with a battery of graded alcohols, immersed in xylene, and cover-slipped with Permount^®^. The number of Ki-67 + cells was quantified by an experimenter blinded to the treatment groups in the dentate gyrus with a Leica DMR light microscope (63x objective lens and 10x ocular lens; total magnification of 630x) while focusing throughout the depth of the tissue section. The number of Ki-67 + cells was corrected by the overall quantified area (mm^2^) of the dentate gyrus, which was measured with a densitometer (GS-800 Imaging Calibrated Densitometer, BioRad). This quantification method allows the comparison among groups of treatment with potential different areas of analysis [34, 37].

### Long-term effects in adulthood

The potential long-term effects of the adolescent cannabidiol treatment on cognition were evaluated with the Barnes maze test in a representative group of rats from Objective 1, which was initially developed to test hippocampal-dependent spatial learning and memory [38]. The Barnes maze equipment used in our study was a circular platform with 18 equidistant holes around the perimeter and a black escape box (target) below one of them, as previously described [33, 39]. The room was filled with visual cues so animals could learn the position of the target box. Moreover, to accentuate the natural agoraphobia of rats, a bright light was used as an aversive stimulus while finding the target. First, rats were allowed to habituate to the maze by placing them in a black start chamber located in the middle of the maze, under a bright light (500 W). After 10 s, the chamber was lifted and rats were allowed to find and enter the black escape box for up to 3 min. On test day, rats were exposed to 3 training trials (separated by 10 min) that finished when the animal entered the target, or after 3 min, when it was manually put into the target box for 1 min. This was followed, 10 min later, by the actual test, used to assess short-term memory, and during which rats were allowed to freely explore the maze for 90 s to find the target box. Moreover, the test was repeated 24 h later to evaluate long-term memory (Fig. 1). The amount of time spent (s) to resolve the maze was used as a measure of spatial working memory performance [33, 39]. Moreover, rats were re-scored in the forced-swim test to ascertain potential long-term effects induced by adolescent cannabidiol exposure through a 5-min videotaped test session as detailed above.

### Data and statistical analyses

Data were analyzed with GraphPad Prism, Version 10 (GraphPad Software, San Diego, CA). Results are expressed as mean values ± standard error of the mean (SEM) with individual symbols for each rat when appropriate [40, 41]. The behavioral and neurochemical effects were evaluated with two-way ANOVAs (independent variables: sex and treatment) at each time point of analysis. Cognitive data was first analyzed for each sex separately through two-way repeated measures ANOVAs, in an attempt to ascertain the potential long-term effects of adolescent treatment, and then at each time-point of analysis including sex as an independent variable. When appropriate, multiple comparisons were performed as detailed in the Figure Legends. The level of significance was set at *p* ≤ 0.05.

## Results

### Antidepressant-like characterization of cannabidiol in adolescence

**Fig. 2:**
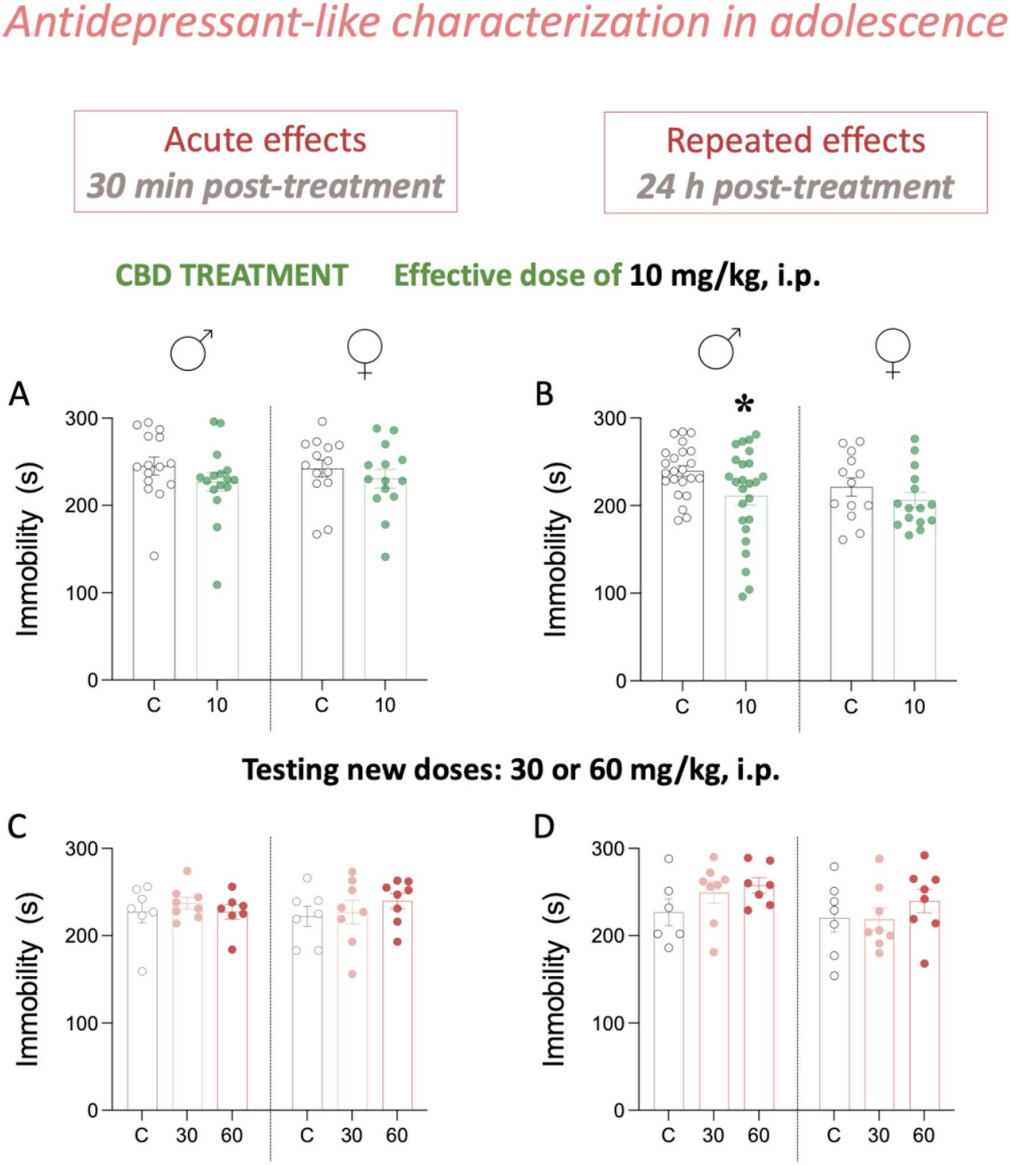
Antidepressant-like effects of cannabidiol in adolescence. **(A-B)** Validating the effective dose of 10 mg/kg of cannabidiol (CBD, *ip*) after an **(A)** acute dose (30 min post-treatment), or a **(B)** repeated treatment (7 doses, 1 dose per day; 24 h post-treatment) as measured under the stress of the forced-swim test (FST) in adolescent rats of both sexes. **(A)** Groups of treatment: Acute effects (30 min post-treatment): male-C (*n* = 15); male-CBD-10 (*n* = 16); female-C (*n* = 14); female-CBD-10 (*n* = 14). **(B)** Groups of treatment: Repeated effects (24 h post-treatment): male-C (*n* = 23); male-CBD-10 (*n* = 25); female-C (*n* = 13); female-CBD-10 (*n* = 15). **(C-D)** Testing new doses (30 or 60 mg/kg, ip.) of cannabidiol (*ip*) after an **C** acute dose (30 min post-treatment), or a **(D)** repeated treatment (7 doses, 1 dose per day; 24 h post-treatment). **(C-D)** Groups of treatment: male-C (*n* = 7); male-CBD-30 (*n* = 8); male-CBD-60 (*n* = 7); female-C (*n* = 7); female-CBD-30 (*n* = 8); female-CBD-60 (*n* = 8). Note that the same rats were assessed following the acute and repeated treatment. **(A-D)** Data represent mean ± SEM of the time spent immobile (s). Individual values are shown for each rat (symbols). Two-way ANOVAs (independent variables: sex, treatment). *Post-hoc* comparisons through Dunnett’s test when appropriate: **p < 0.05 vs. male-C

The first experiment examined the effects induced by a single dose of cannabidiol (10 mg/kg) on the time spent immobile (s) in the forced-swim test in adolescent rats through a two-way ANOVA, with no effects of sex (F_1,55_ = 0.01, *p* = 0.952), treatment (F_1,55_ = 2.05, *p* = 0.158) or sex x treatment interaction (F_1,55_ = 0.10, *p* = 0.750) observed (Fig. 2A). Similarly, no changes were observed in the time rats spent climbing or swimming (Supplementary Fig. 1A). As previously reported in our group [4, 10], when characterizing the repeated administration of 10 mg/kg of cannabidiol during 7 days (1 dose/day), a significant effect of treatment (F_1,72_ = 4.92, *p* = 0.030) was observed. Although this is an overall effect induced by the dose of 10 mg/kg of cannabidiol when combining both sexes, the decrease in immobility was mainly driven by the response in adolescent male rats (-28 ± 12 s, *p = 0.038 vs. C; Fig. 2B), since no differences in immobility were observed in females (cannabidiol vs. control-treated rats). This decrease in immobility was paired with increased time in escaping behaviors (climbing and swimming combined; Supplementary Fig. 1A).

We later tested a couple of higher doses (30 and 60 mg/kg), in an attempt to find an efficacious one for female rats. The results showed no significant effects of treatment following the acute (F_2,39_ = 0.39, *p* = 0.677; Fig. 2C) or the repeated (F_2,38_ = 1.70, *p* = 0.197; Fig. 2D) administration of cannabidiol at the selected doses for male and female adolescent rats, and as measured as the time spent immobile (s) under the stress of the forced-swim test. Moreover, no changes were observed in the time rats spent climbing or swimming (Supplementary Fig. 1C-D).

### Potential drug targets of cannabidiol in adolescence

**Fig. 3:**
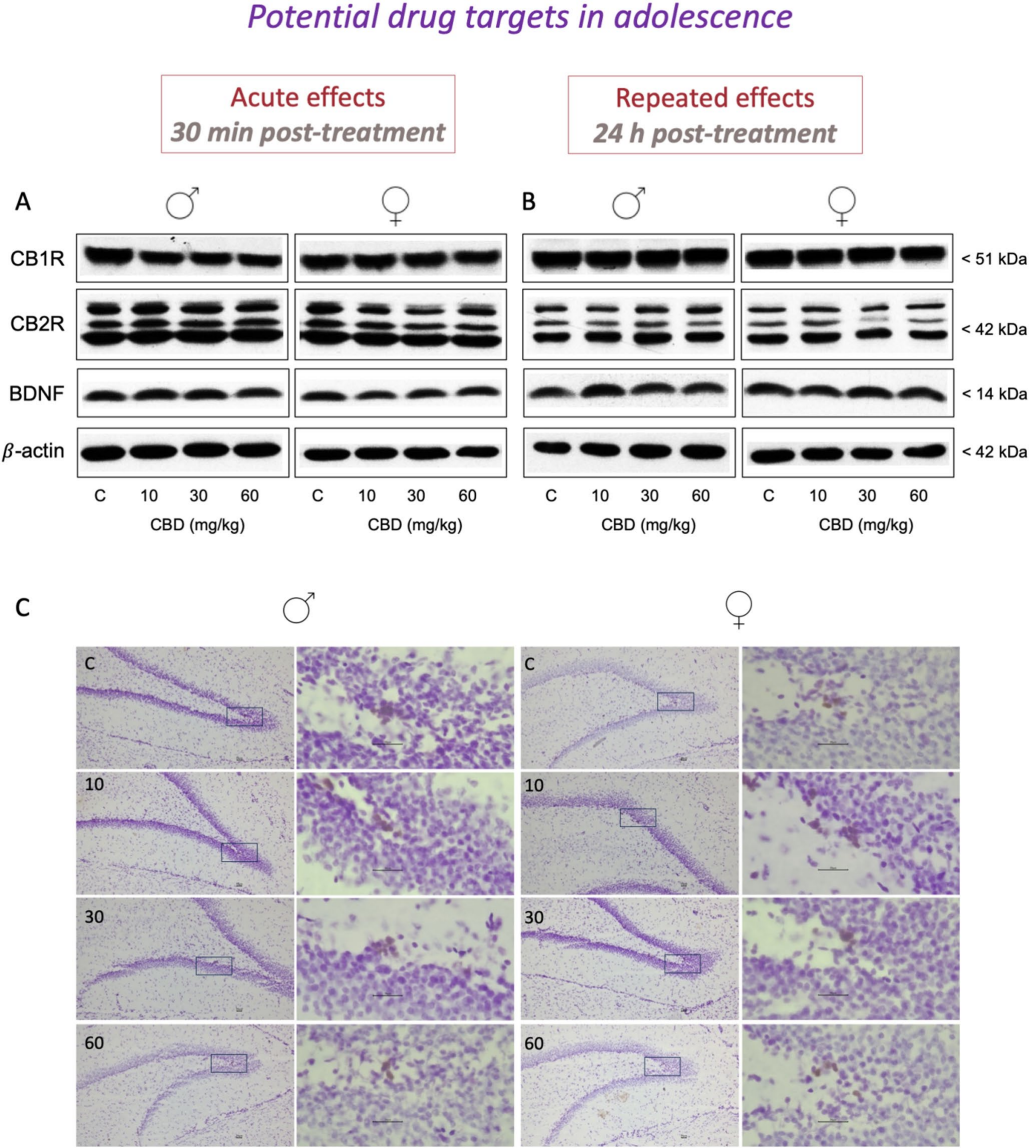
Potential drug targets: neurochemical markers evaluated in the hippocampus of adolescent male and female rats following cannabidiol treatment. Doses tested: 10, 30 and 60 mg/kg, *ip*. The right hippocampus was used to determine target protein content through western blot analyses after an **(A)** acute (30 min post-treatment) and **(B)** repeated (24 h post-treatment) treatment of cannabidiol (CBD). Representative images of selected western blots are shown for cannabinoid (CB) receptor 1 (CB1R) and 2 (CB2R), brain-derived neurotrophic factor (BDNF), and β-actin. **(C)** The left hippocampus was cryostat-cut and used to quantify by immunohistochemistry the number of Ki-67 + cells per area of dentate gyrus (mm^2^), as a marker of cell proliferation, exclusively following the repeated treatment of CBD. Representative images of Ki-67 + cells (brown labeling in the blue granular layer) taken with a light microscope using a 10x objective lens are shown for each treatment condition, together with the magnified window at 63x. Scale bar: 30 μm. The results of the experiments evaluating these drug targets in hippocampal samples of adolescent rats of both sexes treated in adolescence with cannabidiol are summarized in Table 1

When evaluating the effects of adolescent cannabidiol treatment (doses of 10, 30 and 60 mg/kg) on the regulation of several key protein markers (i.e., CB1R, CB2R, BDNF and β-actin as loading control), the main results showed the lack of significant effects of treatment following acute (Fig. 3A) or repeated (Fig. 3B) treatments for all proteins studies (see Table 1). Moreover, the rate of hippocampal cell proliferation was evaluated following repeated treatment with cannabidiol. The results (expressed as % control-male rats) revealed no significant effect of treatment (F_3,39_ = 2.36, *p* = 0.086) when evaluating hippocampal cell proliferation as measured 1-day post-repeated treatment and compared to control rats (Table 1; Fig. 3C).

**Table 1:**
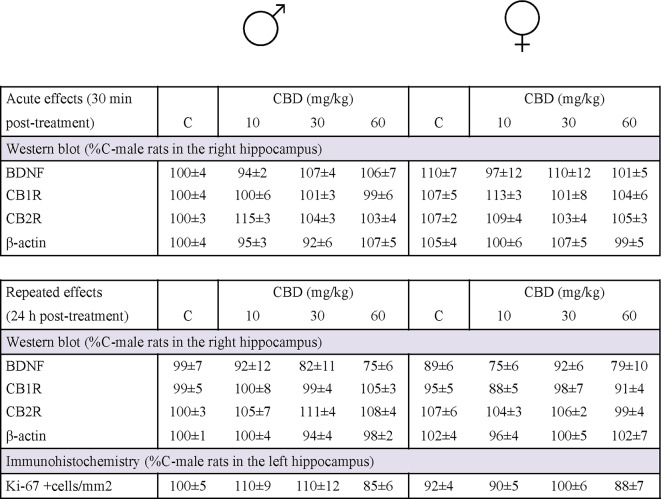
Basal regulation of the potential drug targets evaluated in hippocampal samples of adolescent rats of both sexes treated in adolescence with Cannabidiol (CBD). Doses tested: 10, 30, and 60 mg/kg, *ip*. Acute effects (30 min post-treatment) and repeated effects (24 h post-treatment). The right hippocampus was used to determine target protein content through Western blot analyses, and the left hippocampus was cryostat-cut and used to quantify the number of Ki-67 + cells per area of dentate gyrus (mm^2^) as a marker of cell proliferation

Groups of treatment: Acute effects (30 min post-treatment): male-C (*n* = 5); male-CBD-10 (*n* = 4); male-CBD-30 (*n* = 5); male-CBD-60 (*n* = 5); female-C (*n* = 6); female-CBD-10 (*n* = 6); female-CBD-30 (*n* = 6); female-CBD-60 (*n* = 7). Repeated effects (24 h post-treatment): male-C (*n* = 5); male-CBD-10 (*n* = 5); male-CBD-30 (*n* = 5); male-CBD-60 (*n* = 5); female-C (*n* = 7); female-CBD-10 (*n* = 7); female-CBD-30 (*n* = 7); female-CBD-60 (*n* = 6). Data represents means ± SEM of n experiments per group and expressed in comparison to male controls. No significant differences were observed through two-way ANOVAs (independent variables: sex, treatment). C: control; BDNF: brain-derived neurotrophic factor; CBD: cannabidiol; CB: cannabinoid. Representative images of selected western blots for CB1R and CB2R, BDNF, and β-actin are shown in Fig. 3

### Long-term effects of cannabidiol in adulthood

**Fig. 4:**
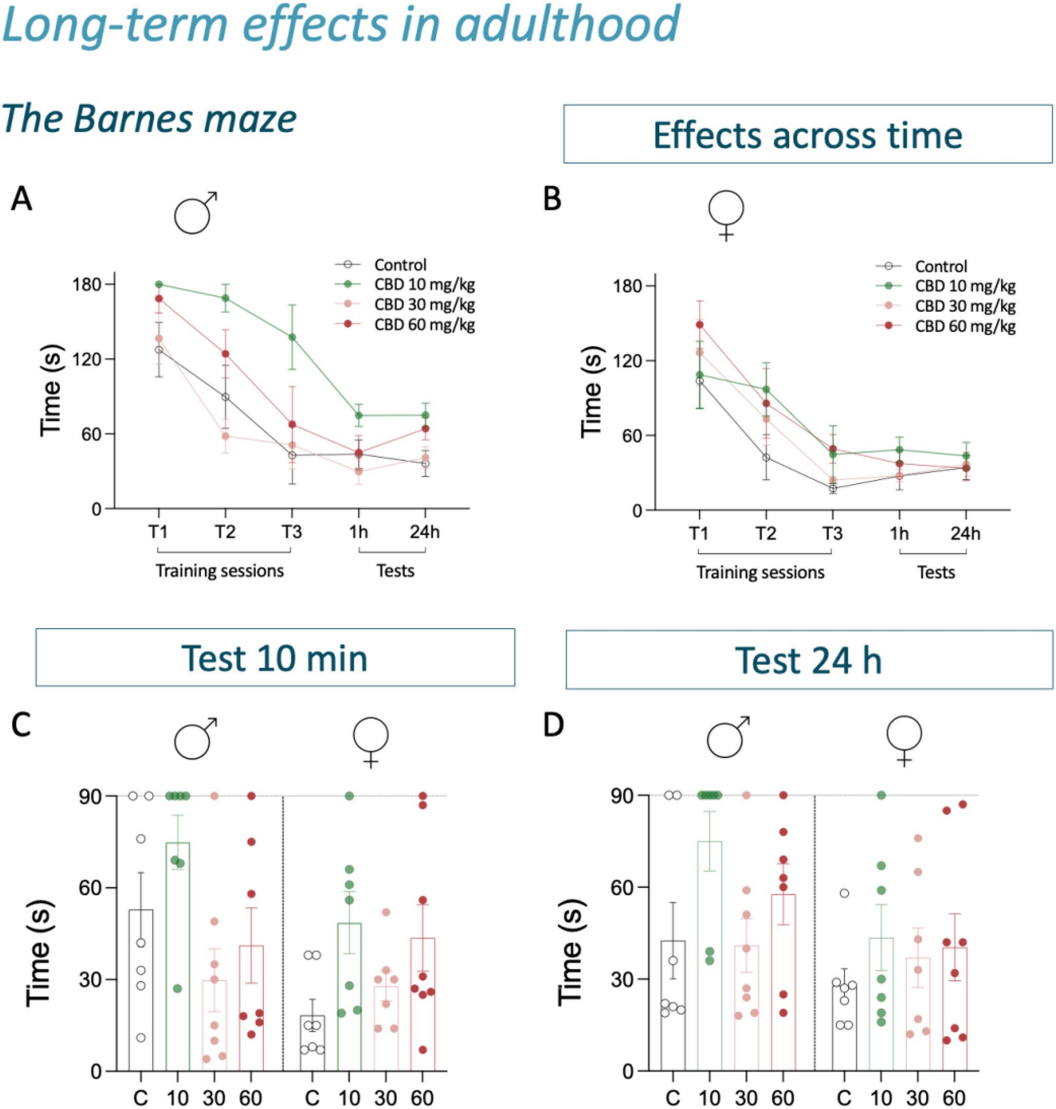
Long-term effects of adolescent cannabidiol treatment on cognitive performance in adult male and female rats. Time spent (s) in 3 consecutive trials (T1, T2, T3) and 2 test sessions (spaced 24 h) to complete the Barnes maze in adult **(A)** male and **(B)** female rats. Data represent mean ± SEM of the time (s) spent to complete the Barnes maze. Note that a time-limit is present for trials (180 s) and test sessions (90 s). Two-way repeated measures ANOVAs were performed to assess the effects across time (independent variables: test day and treatment Group). **(C,D)** Individual values are shown within treatment bars for each rat (symbols) for test sessions 10 min and 24 h. A grid line represents the maximum amount of time available to resolve the maze (90 s). Two-way ANOVAs, with sex and treatment as independent variables, were performed

The long-term effects induced by adolescent cannabidiol treatments on cognitive performance were evaluated in adulthood in the Barnes maze test. In particular, clear sex differences were observed when measuring the time rats needed to resolve the Barnes maze, both during each training session (Fig. 4A vs. Figure 4B; direct comparison not shown in graph) and/or test sessions (test 1 h, effect of sex: F_1,50_ = 4.71, *p* = 0.035, Fig. 4C; test 24 h, effect of sex: F_1,50_ = 5.75, *p* = 0.020, Fig. 4D), with female rats needing significant less time to resolve the maze than males. In conjunction, the rest of the analysis reported no long-term effects driven by adolescent cannabidiol exposure as observed by the lack of sex x treatment interactions for both tests performed in adulthood (10 min: F_3,50_ = 1.71, *p* = 0.178; 24 h: F_3,50_ = 0.64, *p* = 0.592; Fig. 4C and D). Besides the time needed to resolve the Barnes maze, we also quantified the number of errors made while trying to resolve it (data not shown in figures). The results confirmed no major significant long-term negative effects induced by adolescent cannabidiol treatment.

Moreover, adolescent cannabidiol exposure did not change the performance of rats in the forced-swim test in adulthood (data not shown in figures).

## Discussion

The main results validated that the dose of 10 mg/kg of cannabidiol is capable of inducing a moderate antidepressant-like effect in adolescent rats, mainly observed in male rats, while higher doses induced no effects in adolescent rats of both sexes. Moreover, adolescent cannabidiol exposure (acute or repeated) did not alter the neuroplasticity markers evaluated in the hippocampus (i.e., CBR1, CBR2, BDNF, and cell proliferation). Finally, adolescent cannabidiol exposure induced no long-term effects on cognitive performance as evaluated in the Barnes maze or in affective-like responses in the forced-swim test during adulthood. Overall, our data suggest that adolescent cannabidiol treatment produces dose-dependent antidepressant-like effects of small magnitude in rats, without compromising long-term cognition in adulthood.

The potential antidepressant-like beneficial effects of cannabidiol were evaluated under the stressful conditions of the forced-swim test. The results showed that cannabidiol induced an antidepressant-like response which was dose-dependent, mainly observed in male rats, and was measured by a decrease in the time spent immobile in the forced-swim test. In particular, the dose of 10 mg/kg of cannabidiol was capable of exerting an antidepressant-like response of small magnitude in adolescent male rats [4, 10], while being ineffective in females [10]. The higher doses tested (30 and 60 mg/kg) did not induce any changes in male or female rats during adolescence. These results replicated prior data showing that the dose of 30 mg/kg was ineffective in adolescent male rats [4], while extended the results by proving the lack of efficacy induced by the dose of 60 mg/kg in male rats. Moreover, the results showed a complete lack of response in adolescent female rats for all doses tested. A recent study in adolescent male rats showed that while the treatment with 15 mg/kg of cannabidiol did not improve immobility in the forced-swim test, its combination with an also ineffective dose of escitalopram (5 mg/kg) was efficacious, suggesting a synergic effect when combining two drugs [42], an avenue that could be interesting to explore in the context of adolescent female rats. Overall, and together with prior findings [4, 10], cannabidiol is capable of inducing antidepressant-like effects in naïve male rats, while showing no effect in females. These data support a promising therapeutic profile for cannabidiol as an antidepressant of moderate magnitude when administered in adolescence, although mainly for male rats, thus suggesting the need for further studies ascertaining the mechanisms behind the observed sex disparities.

Surprisingly, even though cannabidiol proved sex-specific responses in behavior, these effects did not parallel changes at the level of certain neuroplasticity markers in the hippocampus. In particular, we evaluated whether there was a similar regulation between the antidepressant-like effect observed 1-day post-treatment (i.e., dose-dependent efficacy in adolescent male rats and lack of response in females) and selected hippocampal neuroplasticity markers (i.e., CB1R, CB2R, BDNF, cell proliferation). However, the results indicated that cannabidiol (acute or repeated) did not alter the regulation of CB1R, CB2R, BDNF, or cell proliferation, in the hippocampus of adolescent male and female rats. This either suggested a dysregulation between the behavioral and neurochemical effects in adolescent male rats as measured 1-day post-treatment, or that these markers (traditionally altered by most antidepressants) might not be relevant in the context of cannabidiol’s effects. In fact, there are very limited articles examining the neurochemical effects induced by adolescent cannabidiol exposure in rats of both sexes. While a recent study from our group proved that adolescent cannabidiol exposure did not induce changes in CB1R, CB2R, or BDNF in the hippocampus later on during adulthood [5], no studies seemed to have tested the immediate effects induced in adolescence. Moreover, cannabidiol did not modulate hippocampal neurogenesis, as evaluated by a marker of cell proliferation (Ki-67) and a marker of early neuronal survival (NeuroD) in adolescent rats, in line with previous results [4]. Yet, other studies in the literature do support a role for cannabidiol in inducing hippocampal neurogenesis [20], although in a dose-dependent fashion [43], with lower doses (3 mg/kg) increasing neurogenesis while higher doses (30 mg/kg) decreasing it [44]. However, and contrary to our experimental design, most of the previous studies were centered in adult murine models exposed to a prior stress (reviewed by [23, 45]). Also, similar to our previous [4] and present data, another study showed a disconnection between emotional behavior and cell proliferation regulation following repeated cannabidiol exposure [46], again suggesting that the antidepressant-like effects of cannabidiol might be mediated through different neuroplasticity pathways in naïve animals. Future studies should attempt to find molecular correlates of the antidepressant-like response of cannabidiol during adolescence, as well as the ones behind the observed sex disparities (e.g., [47]). For example, testing how anandamide levels through its pharmacological modulation [48, 49] and/or the activation of 5-HT1A receptors [50, 51] might be driving cannabidiol’s response would be interesting in the context of the present results.

Finally, we aimed at testing the long-term effects driven by a prior repeated exposure to cannabidiol during adolescence, and we did so through evaluating cognitive performance in the Barnes maze test and potential lasting antidepressant-like effects under the stress of the forced-swim test. The results detected clear sex differences in cognitive performance as measured by the time rats needed to resolve the maze, both during training and/or test sessions, with female rats spending significantly less time than males, and thus validating and reinforcing our prior studies [33]. Interestingly, the results proved no long-term impact on cognitive performance in adult male or female rats caused by a prior adolescent treatment with cannabidiol. Moreover, no long-term effects induced by adolescent cannabidiol were detected in the forced-swim test when performed in adulthood, in line with our prior results that proved the return to baseline immobility scores in adulthood following adolescent cannabidiol exposure [10].

To conclude, our data suggest that adolescent cannabidiol treatment produces dose-dependent antidepressant-like effects without compromising long-term cognition in rats. In particular, it reinforces prior studies by including more doses of cannabidiol tested during adolescence, while demonstrating efficacy mainly in male rats. Based on the literature, the combination of another antidepressant with cannabidiol might be a good strategy for attempting to induce efficacy in adolescent female rats, and should be further explored. Since no molecular print could be found to parallel the antidepressant-like potential of cannabidiol in adolescence, future studies should explore other avenues while searching for the specific role of sex hormones in the lack of response in females. Finally, and in line with the results that demonstrate that cannabidiol improves cognitive performance, this study adds to this existing literature by providing long-term results on this topic following adolescent cannabidiol exposure in rats of both sexes.

## Electronic supplementary material

Below is the link to the electronic supplementary material.

Supplementary Material 1

## Data Availability

The datasets generated during and/or analyzed during the current study are available from the corresponding author on reasonable request.
