# Supplementary material for: Adolescent cannabidiol treatment produces antidepressant-like effects without compromising long-term cognition in rats
Source: Pharmacol Rep. 2025 Jun 16;77(4):999–1010. doi: 10.1007/s43440-025-00750-5 (PMC12241299; doi:10.1007/s43440-025-00750-5)
Supplement: Supplementary file 1 — Supplementary Material 1 [file 43440_2025_750_MOESM1_ESM.pdf]

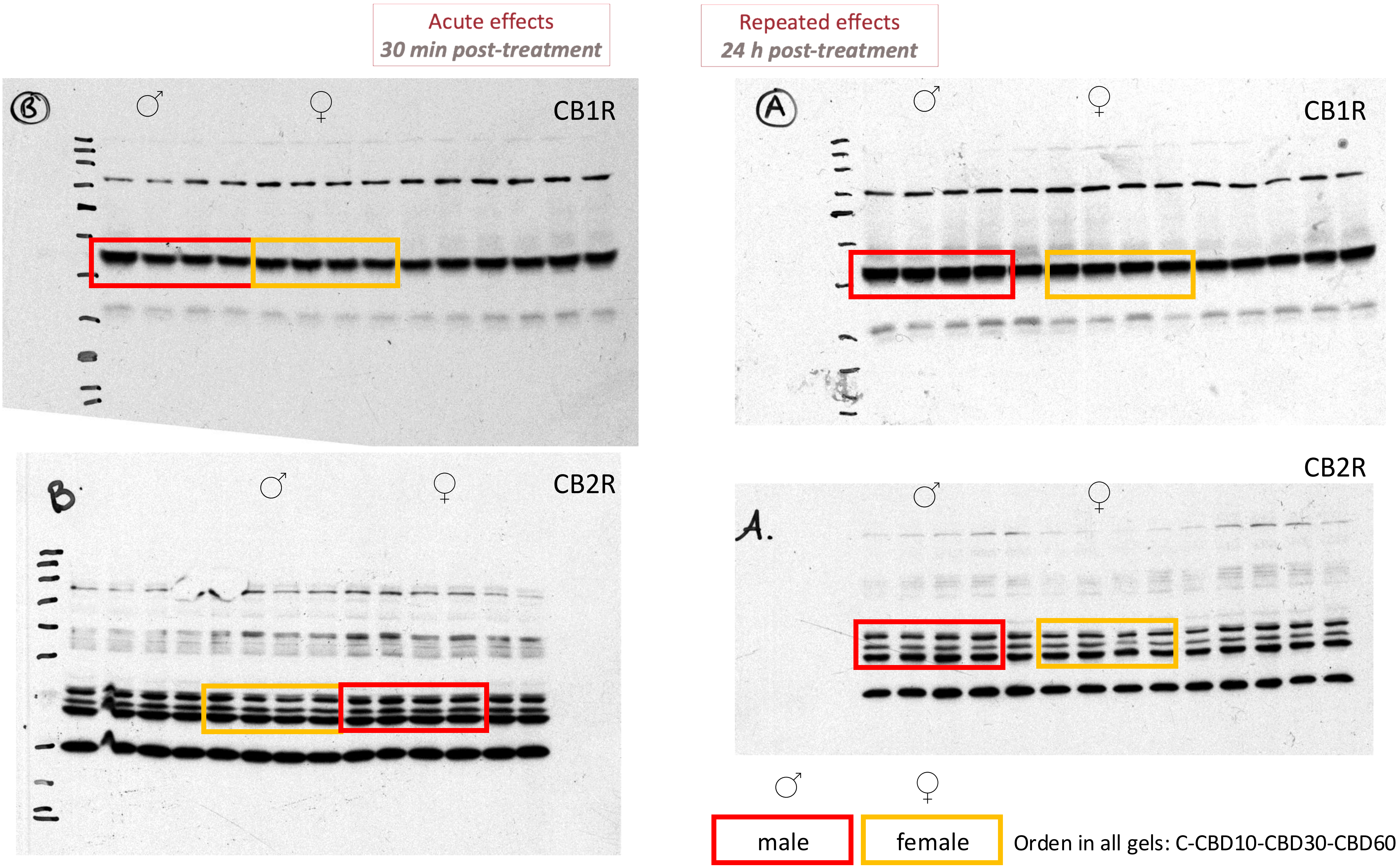
Acute effects
30 min post-treatment
Repeated effects
24 h post-treatment
B
♂
♀
CB1R
A
♂
♀
CB1R
B
♂
♀
CB2R
CB2R
A.
♂
♀
♂
♀
male
female
Orden in all gels: C-CBD10-CBD30-CBD60

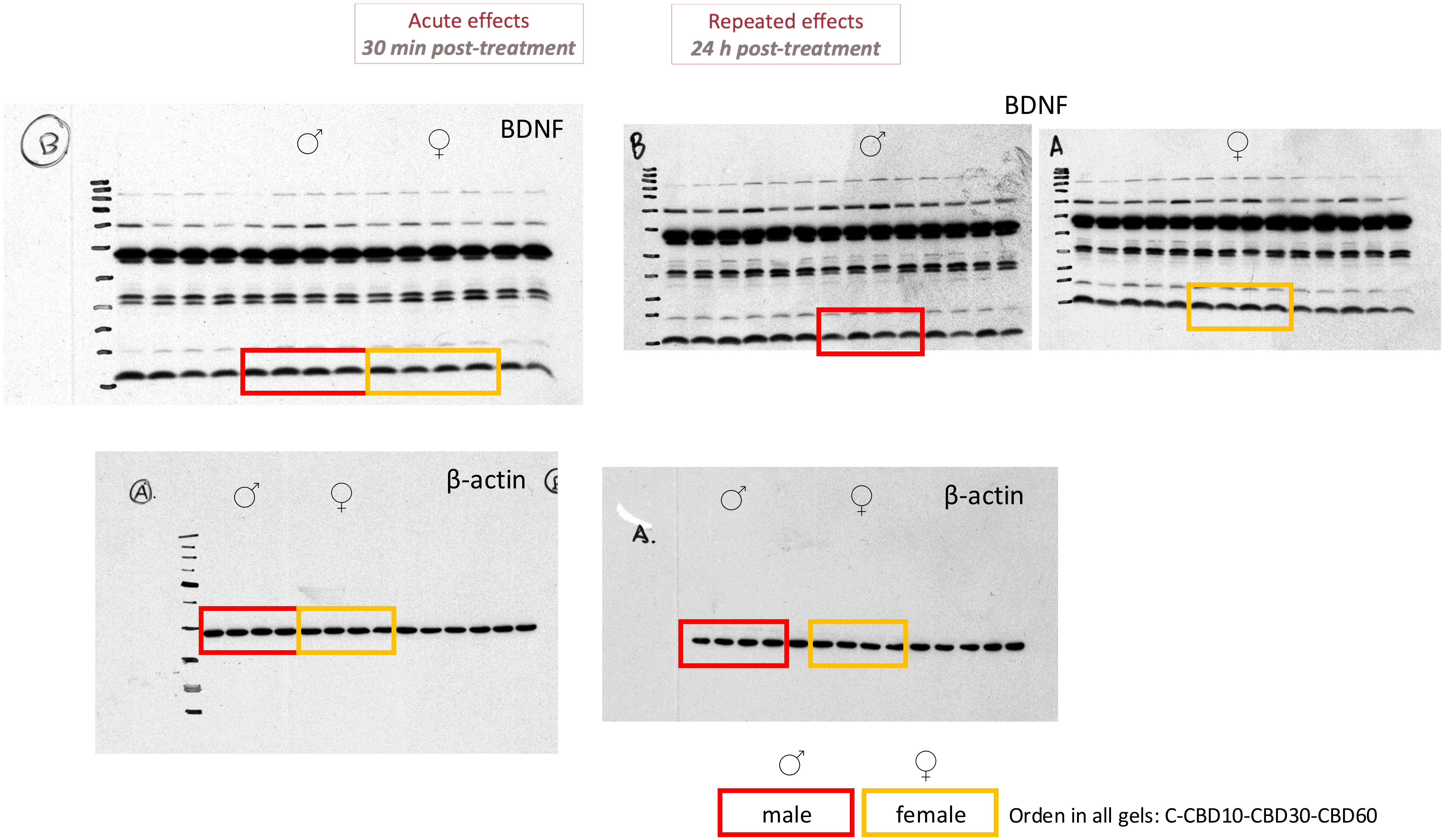
Acute effects
30 min post-treatment
Repeated effects
24 h post-treatment
BDNF
♂
♀
BDNF
β-actin
β-actin
male
female
Orden in all gels: C-CBD10-CBD30-CBD60
